# Hybrid fuzzy inference rules of descent method and wavelet function for volatility forecasting

**DOI:** 10.1371/journal.pone.0278835

**Published:** 2022-12-09

**Authors:** Abdullah H. Alenezy, Mohd Tahir Ismail, Jamil J. Jaber, S. AL Wadi, Rami S. Alkhawaldeh

**Affiliations:** 1 Department of mathematics, College of Science, University of Ha’il, Hail, Kingdom of Saudi Arabia; 2 School of Mathematical Science, Universiti Sains Malaysia, Penang, Malaysia; 3 Department of Finance, School of Business, The University of Jordan, Aqaba, Jordan; 4 Department of Computer Information Systems, The University of Jordan, Aqaba, Jordan; Sant Longowal Institute of Engineering and Technology, INDIA

## Abstract

This research employs the gradient descent learning (FIR.DM) approach as a learning process in a nonlinear spectral model of maximum overlapping discrete wavelet transform (MODWT) to improve volatility prediction of daily stock market prices using Saudi Arabia’s stock exchange (Tadawul) data. The MODWT comprises five mathematical functions and fuzzy inference rules. The inputs are the oil price (Loil) and repo rate (Repo) according to multiple regression correlation, and the Engle and Granger Causality test Engle RF, (1987). The logarithm of the stock market price (LSCS) in Tadawul reflects the output variable. The correlation matrix reveals that there is no collinearity between the input variables, and the causality test demonstrates that the input variables significantly influence the outcome variable. According to the multiple regression, there is a substantial negative influence between Loil and LSCS but a significant positive effect between Repo and output. For the 80% dataset under ME (0.000005), MAE (0.003214), and MAPE (0.064497), the MODWT-LA8 (ARIMA(1,1,0) with drift) for the LSCS variable performs better than other WT functions. In the novel hybrid model MODWT-FIR.DM, each function’s approximation coefficient (LSCS) is applied with input variables (Loil and Repo). We evaluate the performance of the proposed model (MODWT-LA8-FIR.DM) using different statistical measures (ME, RMSE, MAE, MPE) and compare it to two established models: the original FIR.DM and other MODWT-FIR.DM functions for forecasting 20% of datasets. The outcomes show that the MODWT-LA8-FIR.DM performs better than the traditional models based on lower ME (3.167586), RMSE (3.167638), MAE (3.167586), and MPE (80.860849). The proposed hybrid model may be a potential stock market forecasting model.

## 1 Introduction

The stock market collapse is a significant fall in stock prices. Economic crises are typically the cause of stock market collapses. Saudi Arabia’s economy, like other countries, is influenced by global financial crises. The financial crisis of 2007–2008, often known as the “Subprime Mortgage Crisis”, began with the collapse of the United States housing market which eventually brought about the Great Recession. The crisis was a severe global economic depression in the early twenty-first century. It was the worst economic depression since the Great Depression (1929). Between mid-2014 and early 2016, the world economy experienced one of the most significant drops in oil prices in modern history. One of the three biggest price drops after World War II occurred during that time, with prices falling by 70%. However, volatility in the stock exchange market is a statistical measure of the dispersion of close price fluctuations. The term “volatility” refers to the degree of risk or uncertainty connected to the amount of fluctuation in stock prices.. A stock’s value may be more widely distributed throughout a wider range of prices with greater volatility [1]. However, Lower volatility is a sign that the stock’s value is more stable and does not fluctuate significantly. In other words, volatility indicates whether a stock’s price fluctuates dramatically a lot over time (high volatility) or slowly over time (low volatility). The standard deviation in close price from the same stocks or market index is frequently used to measure volatility [1]. To hedge contra unpredictable prices movements, an effective quantitative approach is required to model stock market volatility. The stock market volatility is time-varying, according to previous research. As a consequence, financial econometricians and other practitioners have created a number of time-varying volatility models [2, 3]. In finance and economics fields, the time series prediction approach has a wide range of applications [4, 5]. The methods for time series prediction are classified as statistical or non-statistical. The statistical methods are the autoregressive (AR) and Autoregressive Integrated Moving Average (ARIMA) models. These methods are only applicable for linear time series and are ineffective for nonlinear time series. The non-statistical and soft computing models are fuzzy algorithms and neural networks [6, 7]. Traditional time series models produce accurate forecasts, which is important to highlight. However, because future conditions are uncertain, it is preferable to anticipate a quantity using imprecise values such as fuzzy sets. Time series in the actual world are composed of linear and non-linear components.

A mathematical model called the MODWT is built on five functions; which are Haar, Db, LA-8, BL14, and C6. The literature review indicates that no research has specifically focused on MODWT for modeling and enhancing accuracy in the Tadawul dataset. In terms of the study’s objectives, ranges of comparative studies have been undertaken various methodology of various MODWT functions separately. However, *our contribution is to combine the gradient descent approach (FIR.DM) as a learning method to train the MODWT model that is combined with fitting Fuzzy inference rules in a single particular context or financial market. The gradient descent approach is a backward process that updates the weights of the MODWT model by the error calculated using the predicted and actual values*. The proposed model intends to examine volatility in the Tadawul dataset. The stock index measures the average performance of the Saudi Stock Exchange listed companies. To illustrate the reliability of predictions and the proportion of potential risks, the sources of stock market volatility and variance modeling behavior are provided. As a result, the forecasting accuracy is improved and a new forecasting model is proposed by combining MODWT functions with the FIR.DM model and using a statistical criterion such as the Mean absolute percentage error (MAPE), the mean percentage error (MPE), the Mean error (ME), the Mean absolute error (MAE), and the Root means squared error (RMSE). The proposed model dwells on prior research and enhance stock price volatility forecasting accuracy by combining wavelet function and fuzzy inference rules of descent method. This new model improves stock price volatility forecasting, which is crucial for trading, hedging, and arbitrage purposes.

The structure of this study is as follows. Section 2 shows the previous studies. Section 3 explains the materials and processes. Section 4 discusses the research design and methodology. The empirical findings are analyzed in Section 5. The limitations and directions for future research are discussed in Section 6 followed by the conclusions in Section 7.

## 2 Literature review

Several researchers used different models for time-series forecasting. Chen-Xu and Jie-Sheng [8] used ARMA model to forecast the cash flow of a commercial bank. In order to create an ARMA model to forecast stock returns, Kim investigated the symmetric maximum likelihood (ML) loss function and developed an asymmetric loss function [9]. Similarly, numerous researchers studied ARIMA models for time series predicting [10, 11]. Plenty of studies in time-series forecasting indicate that non-linear models show superior accuracy performance to linear models [12, 13]. As a result, nonlinear models were used for time series forecasting. For instance, Santos and dos Santos Coelho [14] used a non-linear Multilayer Perceptron (MLP) Artificial Neural Network (ANN) with the Takagi-Sugeno fuzzy system and a Radial Basis Function Neural Network (RBFNN) to predict exchange rates. Xiao-Ming and Cheng-Zhang [15] studied 10 ANNs models as base model in AdaBoost approach to predict stock price in the Shanghai Stock Exchange and foreign stock markets. The authors in [16] studied the Haar wavelet and Takagi–Sugeno–Kang (TSK) fuzzy rule-based system to forecast price fluctuations. The TSK fuzzy rule-based model is used with a number of indicators to predict stock prices. According to simulation results, the model effectively predicted the stock price fluctuations in the Taiwan Stock Exchange market with an accuracy up to 99.1%. The authors of [17] suggested a forecasting fusion model that integrated wavelet as a data preprocessing, neural networks, and fuzzy logic. On data from the years 2005 to 2010, they trained the suggested model. The findings indicate that the hybrid model shows superior predicting accuracy to any of the separate models.

Furthermore, the authors in [18] proposed a fuzzy wavelet neural network (FWNN) for forecasting stock prices. The daily dataset of 1000 samples split to 95% from sample for training and the remaining 5% for testing. The simulation results showed that the proposed FWNN system with differential evaluation (DE) training performed better than alternative models. The authors in [4] used ANFIS model, but we used FIR.DM in our proposed model. The model in [4] combined adaptive network-based fuzzy inference system (ANFIS) with five mathematical functions for MODWT to enhance the forecasting accuracy of Saudi Arabia stock exchange (Tadawul). The daily dataset of 2026 samples split to 80% from sample for training and the remaining 20% for testing. The performance of the proposed model better than traditional models.

The TSK model has a special case called fuzzy inference rules with descent method (FIR.DM) [19]. Nomura, Hayashi, and Wakami (1992) used a genetic-algorithm-based method to adjust an input space’s fuzzy partition. They developed a simplified model of fuzzy reasoning, where the real number in the consequent part of the inference rules and the membership functions in the antecedent part are tuned using the descent approach. As a result, the performance of this method is higher than that of a conventional back-propagation type neural network [19, 20]. The form of the membership function of each antecedent fuzzy set and the number of fuzzy if-then rules were determined from numerical data by [20–22]. There are numerous studies on fuzzy inference system learning. Learning approaches that use the steepest descent method (SDM) and vector quantization (VQ) are recognized to be superior to other methods [23]. The learning approaches based on SDM which (1) reduce fuzzy rules to one by one from a significant large number of rules, or create fuzzy rules one by one from any number of rules; (2) determine fuzzy systems by particle swarm optimization (PSO) and genetic algorithm (GA); (3) use single input rule modules (SIRMs) and double input rule modules (DIRMs) approaches, which are fuzzy inference systems with a small number of input rule modules; (4) identify he initial assignment of parameters by self-organization or a vector quantization technique [23].

The volatility is a crucial component for many financial market. it may be used to estimate the risk and reward potential of a certain financial asset. In [24], they employ long short-term memory models (LSTM) and deep neural networks (DNN) as in [25, 26] to predict the volatility of stock indices in US stock market. S&P 500 Index, Dow Jones Industrial Average Index, and NASDAQ Composite Index represent the three samples. The periods are correspondingly 23240, 31096, and 12457 trading days. The findings demonstrate that deep learning models with likelihood-based loss functions are better at predicting volatility than deep learning models with distance loss functions and econometric models, with the LSTM model being the superior model among the two deep learning models with likelihood-based loss functions.Verma [27] predicts the volatility of crude oil using a hybrid models from Bloomberg from 2003 to 2020. He uses Glosten-Jagannathan-Runkle (GJR)-GARCH and generalized autoregressive conditional heteroscedasticity (GARCH) and long short-term memory (LSTM) to create Three novel forecasting models—called GARCH-LSTM, GJR-LSTM, and GARCH-GJRGARCH LSTM. Chen uses the S&P 500 index and WTI oil prices for the period of January 1990 to December 2021 [24]. The nonlinear threshold effect of stock market shock on oil market volatility is captured by the threshold autoregressive regression (TAR) model. According to empirical study, referring to the significant importance of stock volatility’s strong threshold effects for predicting oil volatility.

Macroeconomic factors including unemployment, inflation, and interest rates, gross domestic product, and oil prices have an impact on stock market volatility. In the macroeconomy, the Repo is crucial important factor. The Repo is the monetary policy interest rate, which the central bank uses to lend money to banks for a short period. [28] investigates the impact of repo on the stock market. The other factor is oil price that also refers to the current price of a barrel of crude oil. [29] investigates the impact of oil prices on stock markets. Financial covariates (Repo and Loil) are indeed studied as input factors in our study.

## 3 Methodological issues

The background information for the key ideas in our study is provided in this section.

### 3.1 Autoregressive Integrated Moving-Average (ARIMA) model

One way to assess the strength of a dependent variable (outputs) in relation to other fluctuating variables (inputs) is using a regression analysis called ARIMA model. The purpose of the model is to estimate financial market behaviour by looking at the differences between values in a series rather than actual values. The ARIMA model is a combination of an auto-regressive (AR) model, an Integrated (I) model, and a Moving Average (MA) model. A model in which a changing variable regresses on its own lagged, or prior, values is known as an AR model. The I model denotes the differencing of raw observations in order for the time series to stability become stationary. The MA incorporates the dependency between an observation and a residual error from the MA model applied to lagged observations. A time-series *e*_*t*_ which is called a white noise (WN) process, and *X*_*t*_ is called Gaussian process if for all *t*, *e*_*t*_ is iid *N*(0, *σ*^2^). A time-series *X*_*t*_ is said to follow the ARMA(*p*,*q*) model if [30]:

$$\begin{array}{c} X_{t}=\mu +e_{t}+\sum_{i=1}^{p}\phi_{i}X_{t-i}+\sum_{j=1}^{q}\theta_{j}e_{t-j} \end{array}$$ (1)

where *q* and *p* are non-negative integers, *p* represents order of autoregressive part (AR), *q* is defined as order of the first (MA) part and *e*_*t*_ is the white noise (WN) process. An extension of the ordinary ARMA model is the auto-regressive integrated moving-average model (ARIMA(*p*,*d*,*q*)) given by [30]:

$$\begin{array}{c} \phi_{p}\left(B\right){\left(1-B\right)}^{d}X_{t}=\theta_{0}+\theta_{q}\left(B\right)e_{t} \end{array}$$ (2)

where *p*, *d* and *q* denote orders of auto-regression, integration (differencing) and moving average, respectively. Where *ϕ*_*p*_(*B*) = (1 − *ϕ*_1_ *B*−…−*ϕ*_*p*_ *B*^*p*^) is a *p* order polynomial in *B* and *θ*_*q*_(*B*) = (1+ *θ*_1_ *B*+ …+ *θ*_*q*_ *B*^*q*^) is a *q* order polynomial in *B*. *B* is called time lage operator or backward shift, such as (*B*^2^ *X*_*t*_ = *X*_*t*−2_). When *d* = 0, the ARIMA model reduces to the ordinary ARMA model.

### 3.2 MODWT model

The spectral filtering technique known as Fourier transform (FT) has been extensively employed in industrial and scientific applications. FT transfers a group of complex valued functions to another function; which is known as frequency domain. The Discrete Fourier Transform (DFT) is one type of FT that defined as follow [31]

$$\begin{array}{c} X\left(K\right)=\sum_{n=0}^{N-1}X\left(n\right)W_{n}^{k_{n}},K=0,1,\cdots ,N-1 \end{array}$$ (3)

Where X is time series data, $W_{n}=e^{\frac{-2\pi j}{N}}$, and *N* is discrete point. The inverse discrete Fourier transform (IDFT) was also defined as:

$$\begin{array}{c} X\left(n\right)=\frac{1}{N}\sum_{k=0}^{N-1}X\left(k\right)W_{N}^{-kN},n=0,1,\cdots ,N-1 \end{array}$$ (4)

In light of this, FT and inverse FT, which are created using the previous equations DFT and IDFT, respectively, directly rely on DFT and IDFT.

A feature of the Wavelet Transform (WT) is its ability to “Zoom in” on short lived frequency events. While WT localizes in both the frequency (scale) and time (position) domain, FT only localizes in the frequency domain and not the time domain. The original time series data are transformed using the mathematical function WT into a time-scale domain [32]. The WT transforms the period (or frequency) of data without affecting time resolution. The WT can be applied to both stationary and non-stationary data, such as noise removal from time series, trend analysis and forecasting, and detection of abnormal behavior in data. However, the WT is an attractive option for non-stationary data especially stock market data due to its inherent nature. Three types of wavelet transforms are available: maximum overlapping wavelet transform (MODWT), the discrete wavelet transform (DWT), and continuous wavelet transform (CWT). In general, the aspects of these functions are the same. The main difference between DWT and MODWT is that DWT can be used with a specified number of data (number of observations should be 2 raised to the power J) whereas MODWT can be used for any size of data.

Therefore, our focus in this article is on MODWT due to its the most recent approach for overcoming the Fourier Transform’s (FT) [33, 34]. The WT is an extension of FT [35], which is based on sine and cosine functions. WT satisfies the admissibility condition [30]:

$$\begin{array}{c} C_{\varphi }=\int_{0}^{\infty }\varphi \left(f\right)v\frac{\varphi (t)}{f}df<\infty \end{array}$$ (5)

where *φ*(*f*) is the FT that is a function of frequency f, *φ*(*t*). The applications of WT are image analysis and signal processing [32]. It overcomes the problem of FT, especially when dealing with time, space, or frequency.

The following two WT types are according to Eq 6, the mother wavelet defines the high-frequency (detailed data) components and the father wavelet describes the low-frequency components (smooth data), with *j* = 1, 2, 3, …, *J* in the J-level wavelet decomposition. The General WT model is summarized as:

$$\begin{array}{c} S_{j,k}=\int \varphi_{j,k}f\left(t\right)dt\;\;\;and\;\;\;d_{j,k}=\int \varphi_{j,k}f\left(t\right)dt \end{array}$$ (6)

where *S*_*j*,*k*_ and *d*_*j*,*k*_ demonstrate the smooth and detailed coefficients respectively, J denotes the maximum scale sustainable by the number of data points and the two types of wavelets stated above, the $\phi_{k,j}=2^{\left(\frac{-j}{2}\right)}\phi \left(t-\frac{2^{j}k}{2^{j}}\right)$ and $\varphi_{k,j}=2^{\left(\frac{-j}{2}\right)}\varphi \left(t-\frac{2^{j}k}{2^{j}}\right)$. The smooth coefficients contain the most important features of the original data whereas the detailed coefficients are used to detect the original data’s main fluctuations. The father wavelets and mother wavelets. It satisfies the following conditions:

$$\begin{array}{c} \int \phi (t)dt=1\;\;and\;\;\int \phi (t)dt=0 \end{array}$$ (7)

Generally, the MODWT has popular transform functions; which are Haar, Daubechies(d4), coiflet (c6), Least Asymmetric (LA8), and the best-localized (bl14). The number of main characteristics of these functions are; the MODWT functions are arbitrary regular and do not have explicit expression except the Haar model.. Moreover, the functions use real numbers, orthogonal, compact and support arbitrary number of zero moments, existing of the scale function, continuous / discrete transformation, exact reconstruction, and fast algorithm. However, the Haar model is symmetry. The LA8 and d4 are Asymmetry, whereas near symmetry is associated with C6 and Bl14.

### 3.3 FIR.DM model

FIR.DM is a particular case within the TSK model. This method is proposed by [21]. The FIR.DM uses simplified fuzzy reasoning where the membership functions in antecedent part and the real number in consequent part of inference rules are tuned by means of the descent method. The learning speed and the generalization capability of this method are higher than those of a conventional back-propagation type neural network [21].

#### 3.3.1 The conventional Fuzzy Inference (FI) model

The conventional fuzzy inference (FI) model using descent method is defined [21]. Let *x* = (*x*_1_, …, *x*_*m*_) and y be input and output variables, respectively, where *x*, *y* ∈ *R*. *R* is the set of real numbers. Then, the rule of simplified fuzzy inference model is expressed as [21].

$$\begin{array}{c} Rule\;i:Ifx_{1}\;isA_{i1}\;and\;x_{j}\;is\;A_{ij}\;and\;x_{m}\;is\;A_{im},\;then\;y\;is\;w_{i} \end{array}$$ (8)

where (*j* = 1, …, *m*) is a rule number, (*i* = 1, …, *n*) is a variable number, *A*_*ij*_ is a membership function of the antecedent part (*x*_1_, …, *x*_*m*_), and *w*_*i*_ is a real number of the consequent part (*y*). A membership value of *μ*_*i*_ of the antecedent part for input *x* is expressed as

$$\begin{array}{c} \mu_{i}=\prod_{j=1}^{m}A_{ij}\left(x_{j}\right) \end{array}$$ (9)

Then, the output of fuzzy reasoning *y* can be derived from the equations:

$$\begin{array}{c} y=\frac{\sum_{i=1}^{n}\mu_{i}*w_{i}}{\sum_{i=1}^{n}\mu_{i}} \end{array}$$ (10)

If Gaussian membership function is used, then *A*_*i*_ *j* is expressed as:

$$\begin{array}{c} A_{ij}\left(x_{j}\right)=exp\left(\frac{-1}{2}{\left(\frac{x_{j}-c_{ij}}{b_{ij}}\right)}^{2}\right) \end{array}$$ (11)

where *c*_*ij*_ and *b*_*ij*_ denote the center and the width value of *A*_*ij*_, respectively [21].

#### 3.3.2 Algorithm of self-tuning

The objective function (E) is dedicated to estimate the inference error between the output that is desired and the output that is inferred based on the self-tuning process by a decent method. Let (*p* = 1, …, *P*) is a number of input variables. The objective of learning is to minimize the following mean square error (MSE) as [21]:

$$\begin{array}{c} MSE=\frac{1}{p}\sum_{p=1}^{p}{\left(y_{p}-y_{p}^{r}\right)}^{2} \end{array}$$ (12)

In order to minimize the objective function *MSE*, each parameter of *c*, *b*, *w* is updated based on the learning rule is expressed by the following formula.

$$\begin{array}{c} z_{i}\left(t+1\right)=z_{i}\left(t\right)-k*\frac{\vartheta MSE}{\vartheta z_{i}} \end{array}$$ (13)

where (*z*_*i*_ = *c*_*i*_ *j*, *b*_*i*_ *j*, *w*_*i*_), *t* is a number of iteration time and *K* is a leaning rate constant. The gradients of the objective function $\frac{\vartheta MSE}{\vartheta z_{i}}$ in Eq 13 can be derived from the Eqs 14 to 16, as following [21]:

$$\begin{array}{c} \frac{\vartheta MSE}{\vartheta w_{i}}=\frac{\mu_{i}}{\sum_{i-1}^{n}\mu_{i}}\left(y-y^{r}\right) \end{array}$$ (14)

$$\begin{array}{c} \frac{\vartheta MSE}{\vartheta c_{ij}}=\frac{\mu_{i}}{\sum_{i-1}^{n}\mu_{i}}\left(y-y^{r}\right)*\left(w_{i}-y\right)*\frac{x_{j}-c_{ij}}{b_{ij}^{2}} \end{array}$$ (15)

$$\begin{array}{c} \frac{\vartheta MSE}{\vartheta b_{ij}}=\frac{\mu_{i}}{\sum_{i-1}^{n}\mu_{i}}\left(y-y^{r}\right)*\left(w_{i}-y\right)*\frac{{\left(x_{j}-c_{ij}\right)}^{2}}{b_{ij}^{3}} \end{array}$$ (16)

The learning algorithm for the conventional fuzzy inference model is shown in the following:

**Algorithm 1** Learning Algorithm of the Conventional Fuzzy Inference Model

**Step1:** The threshold *θ* of inference error and the maximum number of learning time *T*_*max*_ are set. Let *n*_0_ be the initial number of rules. Let *t* = 1.

**Step2:** The parameters *w*_*i*_, *c*_*ij*_, and *b*_*ij*_ are set randomly.

**Step3:** Let *p* = 1.

**Step4:** The input-output data (*x*_1_, …, *x*_*m*_, *y*^*r*^) is inputted.

**Step5:** Fuzzy reasoning is performed for the input data (*x*_1_, …, *x*_*m*_) by using Eqs (9 and 10), The membership value *μ*_*i*_ of each inference rule and the output of fuzzy reasoning y are derived.

**Step6:** Parameters *w*_*i*_, *c*_*ij*_, and *b*_*ij*_ are updated by Eqs (14–16).

**Step7:**

**if** p = P **then**

  go to **Step 8**

**else if** p < P **then**

 go to **Step 4**

 *p* = *p* + 1

**end if**

**Step8:** Let *E*(*t*) be inference error at step *t* calculated by Eq (14).

**if** *E*(*t*)>*θ* and *t* < *T*_*max*_ **then**

 go to **Step 3**

 *t* = *t* + 1

**else if** *E*(*t*) ≤ *θ* and *t* < *T*_*max*_ **then**

 The algorithm terminates.

**end if**

**Step9:**

**if** *t* > *T*_*max*_ and *E*(*t*) > *θ* **then**

 go to **Step 2**

 *n* = *n* + 1

 *t* = 1

**end if**

### 3.4 Accuracy criteria

This section can be used to present the criteria that were applied to a fair comparison between actual value and expected value. Five different types of accuracy criteria have been adopted; RMSE, ME, MAE, MPE, and MAPE. The RMSE, also known as root-mean-squared deviation (RMSD), is a commonly used indicator of the divergences between estimators. It determines the average error the model makes while predicting the outcome of an observation. It is defined as $RMSE=\sqrt{MSE}$, where MSE mentioned in Eq (11) [3, 29]. The MPE is the calculated average of the percentage errors between the forecasted values of a model and the original values of the quantity being predicted. As a percentage, it is typically expressed as follows:

$$\begin{array}{c} MAPE=\frac{1}{n}\sum_{t=1}^{n}|\frac{X_{t}-F_{t}}{X_{t}}| \end{array}$$ (17)

Where *X*_*t*_ is the original value, *F*_*t*_ is the forecasted value and *n* is the sample size. The absolute value in this calculation is summed for every forecasted point in time and divided by the number of fitted points. In addition, *MPE*, *MAE*, and *ME* are defined as:

$$\begin{array}{c} MPE=\frac{1}{n}\sum_{t=1}^{n}\frac{X_{t}-F_{t}}{X_{t}} \end{array}$$ (18)

$$\begin{array}{c} MAE=\frac{1}{n}\sum_{t=1}^{n}\left|X_{t}-F_{t}\right| \end{array}$$ (19)

$$\begin{array}{c} ME=\frac{1}{n}\sum_{t=1}^{n}\left(X_{t}-F_{t}\right) \end{array}$$ (20)

The *RMSE* is also known as root-mean-squared deviation (*RMSD*) that is a frequently used measure of the estimators differences. It measures the average error produced by the model in predicting the outcome for an observation. It is defined as:

$$\begin{array}{c} RMSE=\sqrt{MSE} \end{array}$$ (21)

where MSE mentioned in Eq 12 [4, 36].

## 4 Research design and methodology

**Fig 1:**
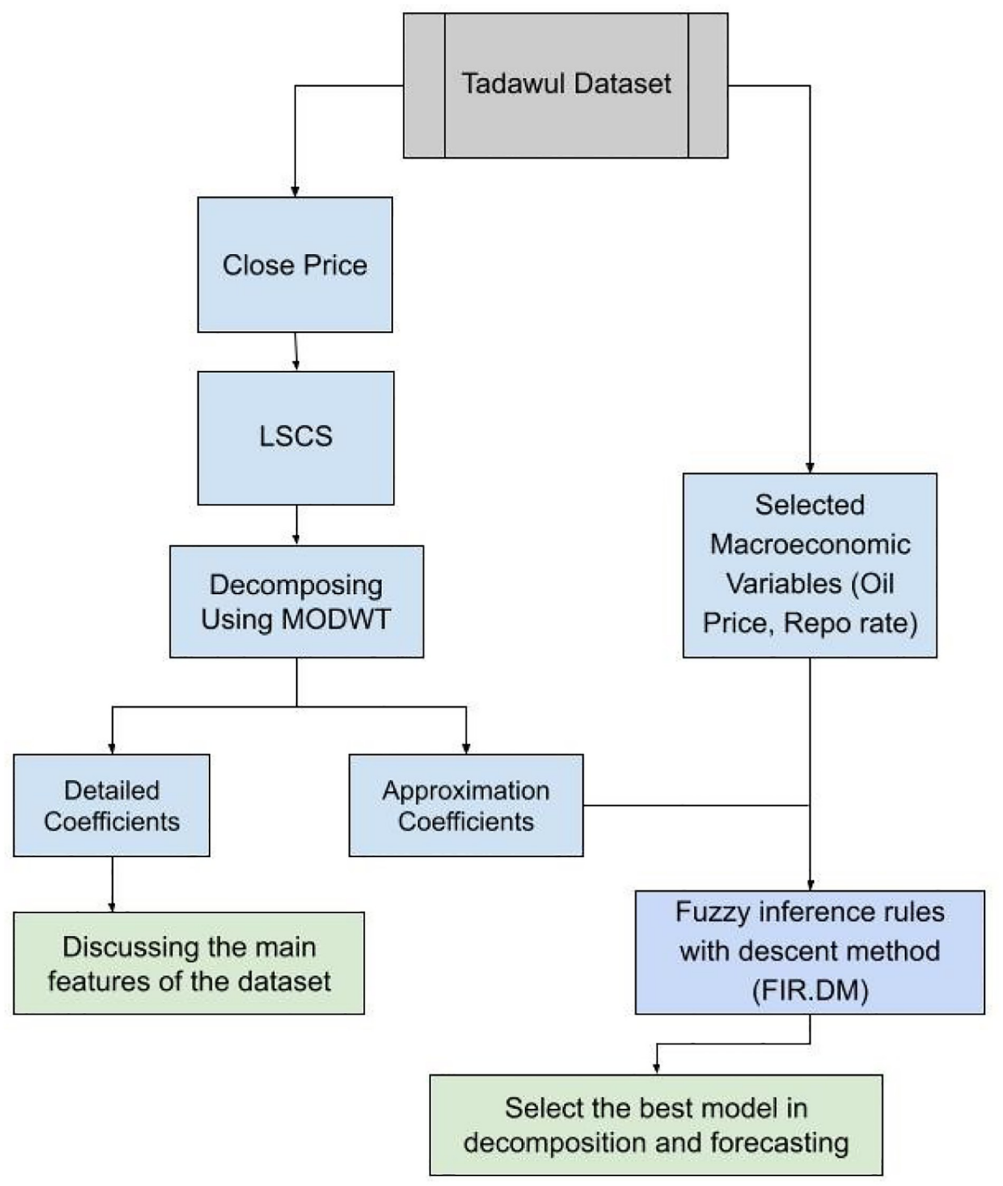
The flowchart of the MODWT with FIR.DM.

The goal of this study is to develop a hybrid model for forecasting closing price data from the Tadawul stock market from 2011 to 2019. The proposed model combines the FIR.DM and MODWT-LA8 models. The Haar, Daubechies (d4), least Asymmetric (La8), and Coiflet (C6) and the best-localized (bl14) are included in MODWT functions. The performance is assessed using the accuracy measure. In addition, the MODWT is used to convert the original data into a time-scale domain. Fig 1 illustrates the different stages of the MODWT forecasting model. It is important to note that the wavelet technique is employed many times while the data pattern was fluctuating. The goal is to minimize statistical error criteria in the data before and after transformation, such as RMSE. Furthermore, the MODWT divides the data into two groups; detail series and approximation series. If the original financial data have significant fluctuation, we used these two groups because they explain the behavior of data.

The proposed hybrid model depends on the robust methodology designed; which include: (1) gathering closing price data from Tadawal, (2) calculating LSCS from closed price data using logarithm standard deviation, (3) decomposing the LSCS data using the MODWT function, which splits the data into two groups: low fluctuated data (approximation coefficient) and high fluctuated data (details coefficient). In addition, Haar, Db, LA-8, C6, and BL14 are five MODWT functions that we used. The main features of the data refer to the approximation coefficient for each function, which is used as an output in the forecasting model, (4) the approximation coefficient (LSCS) for each function is applied with input variables (Loil and Repo) within FIR.DM in new hybrid model MODWT-FIR.DM. Finally, a comparative study is conducted between the best MODWT-FIR.DM model and alternative MODWT-FIR.DM functions as well as traditional models (ARIMA and FIR.DM).

To evaluate the proposed model, we use handout technique of 80% of the original data for model training phase to pick the best effective model, which is further used with other remaining 20% data as test data.

### 4.1 Data description

The stock market (Tadawul) in Saudi Arabia provided the sample data for closing prices. Input for the daily closing prices ranged from August 2011 until December 2019. The sample size of observations is 2026 [30, 33]. Table 1 explores the descriptive statistic of the dataset.

**Table 1:** Descriptive statistic for the Saudi stock market dataset.

	Mean	Std. Deviation	Skewness		Kurtosis	
			Statistic	Std. Error	Statistic	Std. Error
LSCS	6.7489	0.6923	-2.099	0.054	4.263	0.109
Loil	4.2992	0.3535	-0.175	0.054	-1.107	0.109
Repo	0.6963	0.2796	2.006	0.054	22.797	0.109

LSCS refers to the logarithm of standard deviation for closing stock prices, which can be expressed as $\sigma =\sqrt{\frac{\sum_{i=1}^{n}{\left(x_{i}-\bar{x}\right)}^{2}}{n-1}}$, where *x* is closing stock price. Likewise, Repo and Loil represent the repo rate and logarithm of oil prices, respectively.

**Fig 2:**
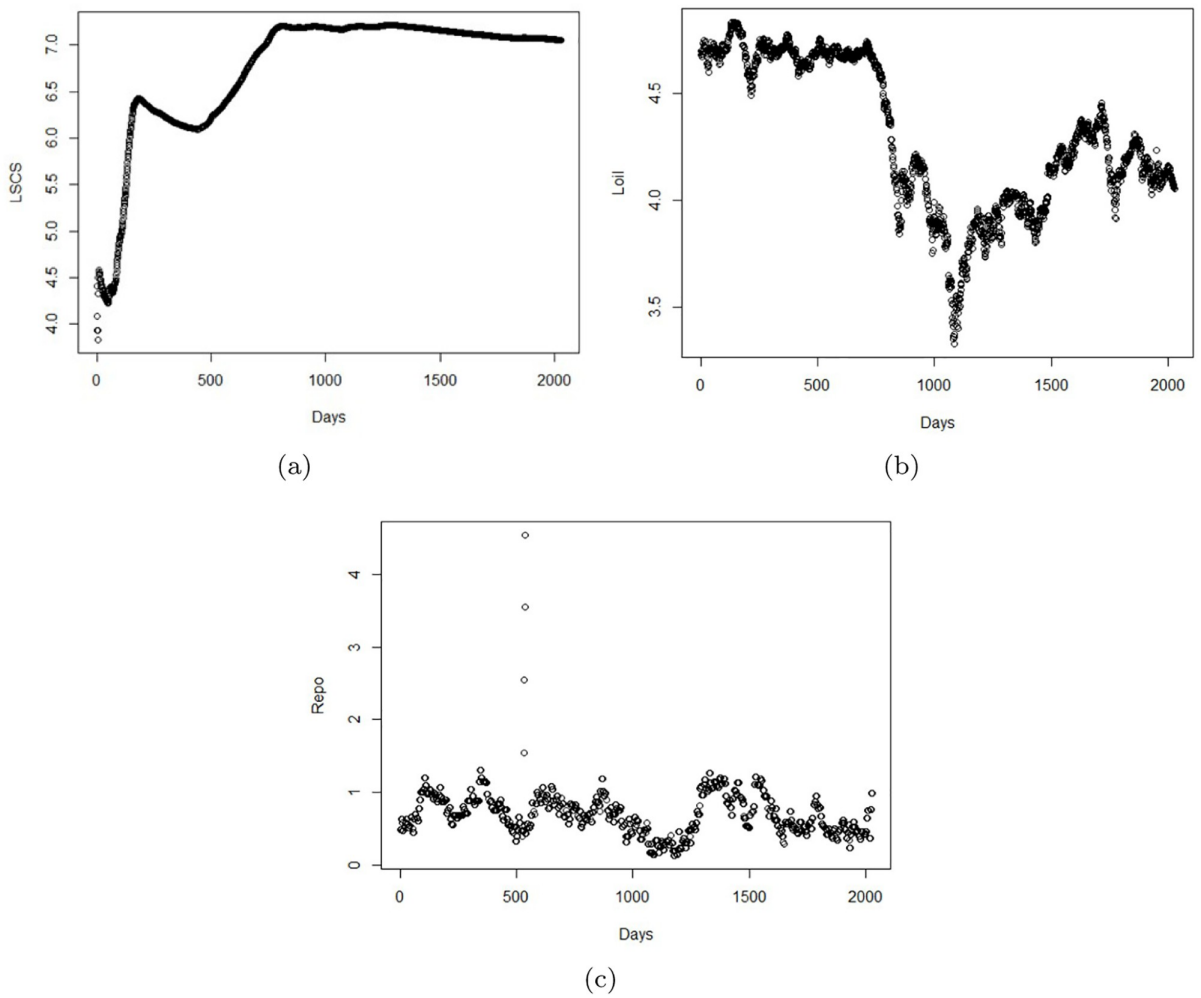
Data description over days (a) LSCS LSCS; (b) Loil Loil; (c) Repo Repo.

The mean and standard deviation of LSCS are 6.75 and 0.69, respectively. The skewness and kurtosis values of LSCS are -2.10 and 4.26, respectively. It should be note that the mean and standard deviation of Repo are 0.70 and 0.28 whereas the skewness and kurtosis values of Repo are 2.00 and 22.80, respectively. The mean and standard deviation of Loil are 4.30 and 0.35, correspondingly. The skewness and kurtosis values of Loil are -0.18 and -1.11, respectively. The graph representation of the datasets over days are shown in Fig 2.

### 4.2 Endogeneity issues

This section discusses how to choose appropriate variables by eliminating multicollinearity, multiple regression analysis, and causality test.

#### 4.2.1 Correlation

In this section, we carefully picked independent variables from various other variables, which are eliminated based on certain test. First, we removed variables as a result of multicollinearity among independent variables as shown in Table 2. The absence of perfect multicollinearity, an exact (non-stochastic) linear relationship between two or more independent variables, is generally referred to as no multicollinearity. We extracted some variables from independent variables according to the strong relation with other independent variables. Table 2 gives the significant correlations between the independent and the dependent variables.

**Table 2:** The significant correlations between the selected variables.

	LSCS	Repo	Loil
LSCS	1		
Repo	-.149**	1	
	-.673**	.327**	
Loil			1

#### 4.2.2 Engle and granger causality test

The Engle and Granger test uses co-integration to illustrate the causal relationships. It produces residuals (errors) depending on static regression. Using an Augmented Dickey-Fuller test or another similar test, the test uses residuals to see whether unit roots are available. If the time series is co-integrated, the residuals would be almost stationary [37]. $d\left(Y_{t}\right)=c+\sum_{i=1}^{n}\beta_{n}*=\left(Y_{t-i}\right)+\sum_{i=0}^{n}\alpha_{n}*=\left(Y_{t-i}\right)+-ECT_{t-1}+\epsilon_{t}$ where *Y* is the dependent variable, *X* is the independent variable set, ECT is the word for error correction where as *β*, *α*, and are the parameters. The null hypothesis for the Engle Granger test (H0: There is no cointegration) is rejected if *ϕ* is is negative or higher than 1.96, the Engle Granger test’s null hypothesis (*H*_0_: There is no cointegration) is rejected. The null hypothesis is rejected, indicating that independent factors influence dependent variables.

In Table 3, an explanation of the dependent and independent variable Engle and Granger test is provided. According to the results, there is co-integration between independent and dependent variables at a significant level of 5%. This result almost implies that the independent factors are the ones responsible for the dependent variable.

**Table 3:** Engle and granger causality test.

Variables	Repo	Loil	All
Estimate	-0.006	-0.0061	-0.007
SE	0.0014	0.0013	0.0016
t-stat (*ϕ*)	-4.201	-4.71	-4.244
Sig.	2.77E-05	2.65E-06	2.30E-05
Causality	Causality	Causality	Causality

#### 4.2.3 The regression analysis

Regression analysis is important for statistics because it may help researchers identify the variables that are most important, the ones they can ignore, and the relationships between those variables. Forecasting and determining the causal connection between variables are both done using also regression analysis. LSCS is the dependent variable, whilst Repo and Loil are the independent variables that are employed to forecasted LSCS in this study. In Table 4, the multiple regression analysis is presented. The Repo rate and Loil are important at 1 percent. Additionally, R-square and modified R-square are close to 45%, which indicates that the independent variables may contribute for 45% of the dependent variable, The linear regression model is more appropriate to the data, according to an F-statistic of 1 percent.

**Table 4:** The multiple regression.

	Intercepte	Repo	Loil	R-square	Adjusted R-square	F-statistic	p-value
Estimate	12.427	0.264	-1.368				
Std. Error	0.147	0.047	0.036	0.4552	0.4546	760.3	< 2.2e-16
t value	84.41	5.683	-38.424				
Pr(> |*t*|)	<2E-16 ***	1.54E-08 ***	< 2E-16 ***				

(Hint: 1% “***”, 5% “**”, and 10% “*”)

**Fig 3:**
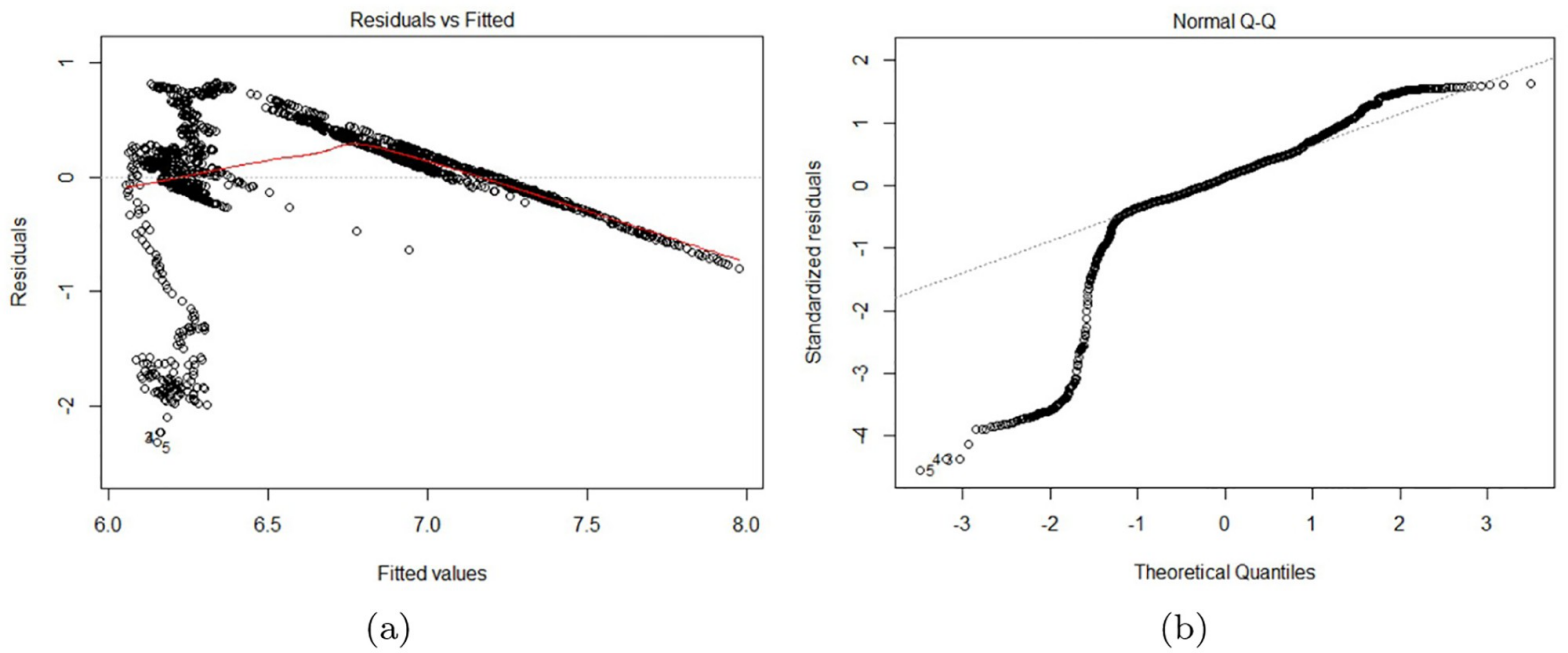
The multiple regression analysis (b) Loil Residuals vs Fitted; (c) Repo Normal Q-Q.

Oil prices and volatility risk, which calculates the standard deviation of closing stock prices, have a negative association. This suggests that the stock market volatility will decrease as oil prices rise. The repo rate, on the other hand, has a favorable association with volatility risk. This suggests that the volatility risk would rise by around 26% as a result of the increase in repo rate. In Fig 3, the residuals approximately match with the diagonal line. These residuals seem to be dispersed normally.

## 5 Results and discussion

**Fig 4:**
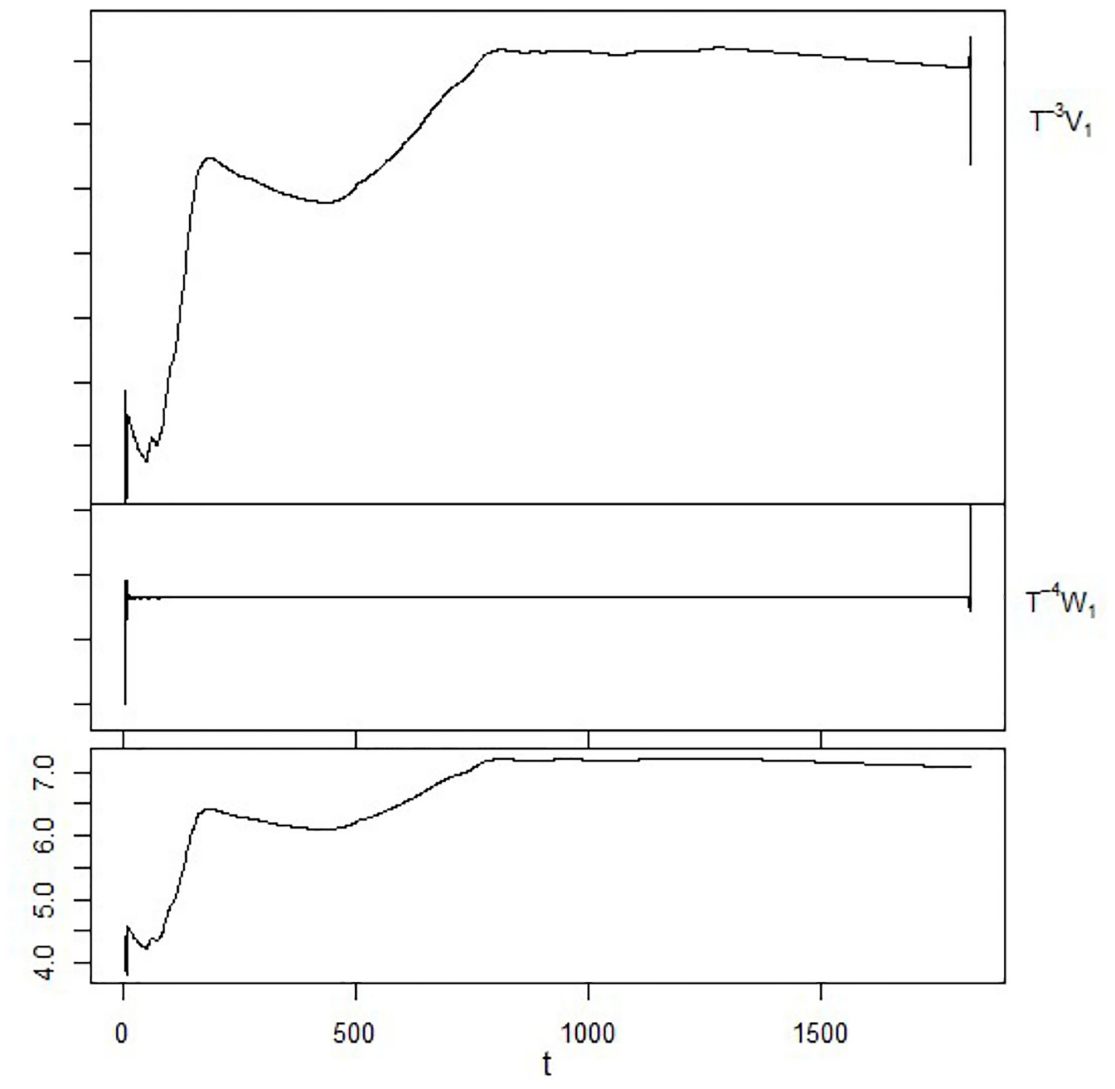
Decomposing the data using MODWT based on La8 function.

The data from Tadawul’s closing prices are examined in this study. It is selected for many reasons. The history of stock market volatility in emerging economies is interesting. The Saudi market serves as an illustration of how enormous volatility may result from informational imbalances, irrational trading, and inexperienced financial analytics. Additionally, investors from nations outside of the Gulf Cooperation Council (GCC) are not permitted to purchase Saudi Arabian securities. The biggest city in the Middle East is Tadawul. Therefore, the volatility data is decomposed using MODWT with La8 function as shown in Fig 4.

In the beginning, the closing price data have been processed with logarithm standard deviation to find LSCS. Next, MODWT has been used to decompose the LSCS data using R-statistic software. The MODWT technique split the LSCS data into two groups, namely, low fluctuated data(approximation coefficient) and high fluctuated data(details coefficient). The main features of the data refer to the approximation coefficient for each function, which is used as an output in the forecasting model. Haar, Daubechies (Db), least Asymmetric (LA-8), Coiflet (C6), and the best-localized function (BL14) are the five MODWT function which have been used. As a result, LA-8 is the most efficient MODWT function (see Table 5). Fig 4 shows the best MODWT performance when using the LA-8.

**Table 5:** The WT function of output variable for 80% dataset.

WT Function	Haar	Db4	LA-8	BL-14	C6
	ARIMA(0,1,1) with drift	ARIMA(0,1,0)	ARIMA(1,1,0) with drift	ARIMA(1,1,0) with drift	ARIMA (1,1,0) with drift
ME	0.000396	0.000576	**0.0000053**	0.0000096	0.000033
MAE	0.004131	0.004708	**0.003214**	0.003294	0.003315
MAPE	0.08893	0.09548	**0.064497**	0.065578	0.06605

The MODWT-based decomposition is an effective technique for displaying data fluctuations and significance levels. The decomposition levels can be carried out by the *WT* using the formula, according to the WT mechanism: *X* = *TV*1 + *TW*1 where the original signal is referred to as *X*. The next component consists of one level of approximation (*TV*1) that shows the plot of the transformed data approximation coefficients. The following parts of *TW*1 reflect the level of detail, whereby *TW*1 is the plot of the first level of the coefficients of detail, so the fluctuation can be explained by this level. It’s interesting to note that 1620 samples out of a total of 2026 are used to represent 80 percent of the data.

Tadawul has experienced several fluctuations from 2011 until 2019. In 2011, the general index of the stock exchange market declined to 6417.7 points, but it climbed to 8535 points in 2013. To increase the market’s efficiency and efficacy, market management changed the trading mechanism from (SAXESS) to (X-Stream INET), and an interactive multi-user system (IFSAH) was designed [38]. One of the challenges that confronted different economies around the world is the fluctuation of stock prices. Both the domestic and foreign economies have been effected on Tadawul. As a result, the effects of financial crises in other economies are transferred to the domestic economy. Consequently, the global financial crisis in 2008 hit Tadawul heavily [39].

The results of the proposed models applying the first 80% of the dataset are shown in Table 5. The original LSCS dataset are provided by MODWT models. MODWT (LA-8) is the best model based on the comparison because it has a minimum value of 0.0000053, 0.003214, and 0.064497, respectively, for ME, MAE, and MAPE. The FIR.DM model was constructed using Repo and Loil as input variables, whereas the MODWT (LA-8) model used LSCS as an output variable.

Table 6 shows The result of forecasting WT Models with FIR.DM. The table explain the remaining 20% of the original and transformed data with the same proposed models in order to validate our results. The best model is ARIMA MODWT (LA-8) with FIR.DM because it has the lowest ME, RMSE, MAE, and MPE. Similar to the training phase, LSCS is used as output variable whereas Repo and Loil are used as input variables by MODWT to construct FIR.DM and FIR.DM + MODWT models.

**Table 6:** The WT function of output variable for 20% dataset.

Models	FIR.DM	FIR.DM+WT(Haar)	FIR.DM+WT(d4)	FIR.DM+WT(la8)	FIR.DM+WT(bl14)	FIR.DM+WT(c6)	FIR.DM+ARIMA direct
ME	3.2555060	3.2024690	3.2814411	**3.1675864**	3.2461338	3.2130600	3.2555060
RMSE	3.2555570	3.2025209	3.2814918	**3.1676389**	3.2461850	3.2131118	3.2555570
MAE	3.2555060	3.2024690	3.2814411	**3.1675864**	3.2461338	3.2130600	3.2555060
MPE	85.0132386	82.4858285	86.2748067	**80.8608493**	84.5615382	82.9849967	85.0132386

## 6 Limitations and directions for future research

This research used MODWT-LA8-FIR.DM to improve volatility prediction of Tadawul (Saudi Arabia’s stock exchange dataset). There are several limitations of the research. (1) we merely use oil price and repo rate as input variables. In the future, we intend to address this restriction utilizing different macroeconomic variables. (2) we consider the Tadawul dataset, intending to expand the experiments to include data from other stock exchange markets, including the New York Stock Exchange (NYSE), National Association of Securities Dealers Automated Quotations (NASDAQ), Shanghai Stock Exchange (SSE), and Hong Kong Stock Exchange (HKSE). (3) the limited daily data are selected from 2011 to 2019 without considering the COVID-19 period. In future work, we will consider COVID-19’s daily stock price data in our upcoming research.

## 7 Conclusion

We proposed a hybrid model (MODWT-LA8-ANFIS) with gradient decent learning approach. The model is applied on the Saudi Arabian stock exchange (Tadawul) to predict the closing price. According to correlation, multiple regressions, and the Engle and Granger Causality test, we picked the oil price and the repo rate as input values. The result found that the input variables had a weak correlation(*r* < 50%). On the other hand, there is a strong correlation (*r* ≥ 50%)between the price of oil and the output variable (closing price). Furthermore, the Engle and Granger Causality test shows that the input variables have been causally related to the output variables. The multiple regression test is used to determine whether the impact is significant. Consequently, the input variables have a level of significance at 5%. The output variable is based on 2026 observations from Tadawul between October 2011 and December 2019. The Saudi Authority for Statistics and the Saudi Central Bank provided the study’s input variables. The MODWT technique divides variables into approximation and detailed coefficients. Haar, Daubechies (Db), least Square (LA-8), Best localization (BL14), and Coiflet (C6) are the five MODWT functions. As a result, the output variable is divided into two parts: details coefficient (highly fluctuated data) and approximation coefficient (lowly fluctuated data) which consists of the main features of data. Our MODWT-LA8- FIR.DM model is built using approximation coefficient data (MODWT-LA8) and input variables. Statistical criteria tests were used to assess the MODWT-LA8-FIR.DM, including mean error (ME), root mean squared error (RMSE), and mean absolute percentage error (MAPE). Traditional models (ARIMA and FIR.DM models) were compared to the MODWT-LA8-FIR.DM model. Traditional models are less accurate than the MODWT-LA8-ANFIS. As a result, the new forecasting model proposed may be applied to other foreign stock markets.
